# Postoperative Pain after Endodontic Treatment of Asymptomatic Teeth Using Rotary Instruments: A Randomized Clinical Trial

**DOI:** 10.7508/iej.2016.01.008

**Published:** 2015-12-24

**Authors:** Shahriar Shahi, Vahideh Asghari, Saeed Rahimi, Mehrdad Lotfi, Mohammad Samiei, Hamidreza Yavari, Sahar Shakouie, Saeed Nezafati

**Affiliations:** a *Dental and Periodontal Research Center, Department of Endodontics, Dental School, Tabriz University of Medical Sciences, Tabriz, Iran; *; b * Department of Endodontics, Dental School, Tabriz University of Medical Sciences, Tabriz, Iran; *; c * Department of Oral and Maxillofacial Surgery, Dental School, Tabriz University of Medical Sciences, Tabriz, Iran*

**Keywords:** Irreversible Pulpitis, Flare-Up, Postoperative Pain, ProTaper, RaCe, Root Canal Treatment

## Abstract

**Introduction:**

The aim of the present study was to compare the effect of two different rotary instruments on postoperative pain in teeth with asymptomatic irreversible pulpitis.

**Methods and Materials:**

A total of 78 mandibular first and second molars were divided into two groups (*n*=39) and their root canal preparation was carried out with either RaCe or ProTaper rotary instruments. All the subjects underwent one-visit root canal treatment and the severity of postoperative pain was evaluated using visual analog scale (VAS) at 4-, 12-, 24-, 48- and 72-h and 1-week intervals. In addition, the need for taking analgesics was recorded. Data were analyzed with the repeated-measures ANOVA and the Mann-Whitney U test was used for two-by-two comparison. Statistical significance was set at 0.05.

**Results:**

Comparison of mean pain severity between the two groups at various postoperative intervals did not reveal any significant differences (*P*=0.10). The difference in amount of analgesics taken by each groups was not statistically significant (*P*=0.25).

**Conclusion:**

There were no significant differences in the postoperative pain reported between the two groups; which indicates the clinical acceptability of both systems.

## Introduction

Incidence of endodontic postoperative pain subsequent to endodontic treatment ranges from 1.4 to 16% [1]. The common factors contributing to postoperative pain and discomfort after root canal treatment include inadequate instrumentation, extrusion of irrigation solutions, extrusion of intracanal dressing, traumatic occlusion, missed canals, preoperative pain, periapical pathosis and extrusion of apical debris [2]. Evidence show that apical extrusion of infected debris during chemomechanical instrumentation is the main etiologic factor for periapical inflammation and postoperative pain [3]. 

Several factors affect the extrusion of debris, including the irrigation protocol [4], the final apical size [5], the time spent on root canal instrumentation [6] and the technique employed for it [7] and the instrument design [8]. All of the instrumentation techniques result in apical extrusion of debris to some extent, no matter how much caution is given to confine the preparation to the apical terminus. However, there are claims that some rotary techniques minimize extrusion of debris more than others [3].

The majority of recently introduced nickel-titanium (NiTi) rotary instruments result in minimal debris extrusion compared to the stainless-steel hand K-files, which is attributed to their rotary action, Archimedes’ screw effect and copious irrigation associated with these instruments [9]. Two of the most commonly used rotary systems are RaCe (FKG Dentaire, La-Chaux-de Fonds, Switzerland) and ProTaper (Dentsply Maillefer, Ballaigues, Switzerland) systems which are mainly used with the crown-down single-length technique. 

RaCe instruments consist of two grooves followed by one straight grooves-free area along the file, for accumulation and evacuation of debris, which results in a decrease in screw-in effect, along with enlargement of the coronal area of the root canal. This design also provides a passageway for debris to escape from the root canal that reduces the apical extrusion of debris, resulting in less severity of postoperative pain [10].

ProTaper instruments possess a convex triangular cross-sectional design and flutes along the file that are combined with variable tapers along the file shaft. It has been claimed that such a design is more effective in cutting dentin [11].

Ahmed *et al.* [12] reported no statistically significant differences in the severity of postoperative pain among the patients treated with ProTaper files or manual step-back technique. Aqrabawi and Jamani [13] demonstrated no statistically significant difference in the postoperative pain among the patients whose teeth were treated with ProTaper or K-Flexo hand files at any time period. Based on a study by Nekoofar *et al*. [14] the postoperative pain was significantly lower in patients treated with ProTaper rotary instruments compared with the WaveOne reciprocating single-file system.

Tasdemir *et al*. [15] showed that ProTaper and Mtwo Rotary instruments extruded more debris than RaCe files and Garlapati *et al*. [16] reported that Mtwo and ProTaper instruments exhibited significantly more apical extrusion of bacteria than RaCe. Furthermore, Tanalp *et al*. [17] showed that ProTaper files extruded significantly greater amounts of debris in comparison to other continuous rotary techniques.

Since there is no clinical trial available to compare postoperative pain after the endodontic treatment using RaCe and ProTaper rotary instruments, the present study was undertaken to compare the severity of postoperative pain after endodontic treatment of the first and second mandibular molars with asymptomatic irreversible pulpitis using these two common systems.

## Materials and Methods

This study was approved by the Ethics Committee of Tabriz University of Medical Sciences (Grant No.: 1394.47) and was registered at Iranian Registry of Clinical Trials (Registration ID: IRCT201503035141N3).

After conducting a pilot study and considering *α*=0.05 and power of 80%, the sample size was estimated to be 78 (*n*=39). All the subjects were treated in the Postgraduate Clinic of the Department of Endodontics, Faculty of Dentistry, Tabriz University of Medical Sciences, Tabriz, Iran.

All patients had a first or second asymptomatic mandibular molar diagnosed with irreversible pulpitis and a normal periapical radiographic view. Before initiation of treatment, the whole procedural steps were explained to the patients. Then the patients signed an informed consent form. 

The inclusion and exclusion criteria are shown in Table 1. The pulpal and periradicular status was assessed using vitality thermal and electric pulp tests (Diagnostic Unit; Sybron, Orange, CA), palpation and percussion in all the patients. The clinical diagnosis of asymptomatic irreversible pulpitis was established by the presence of increased or prolonged response to cold testing with Green Endo-Ice (1,1,1,2-tetrafluoroethane; Hygienic Corp, Akron, Oh, USA) and the presence of deep caries that extended to pulp space on radiographic view, without any symptoms. Periodontal charting was also carried out. Periapical radiographs were taken using a digital radiographic technique (Kodak RVG 5100 Digital Radiography System, Ontario, Canada) and saved.

Patients were randomly treated by choosing a packet in which the type of the instrument was written. The patients were unaware of the types of the instruments used for endodontic treatment; therefore, the study was considered a single-blind one. All the subjects underwent standard IANB anesthesia and long buccal infiltration with 2% lidocaine containing 1:80000 epinephrine (Darupakhsh, Tehran, Iran) with a side-loading cartridge syringe (Dena Instruments, Forgeman Instruments Co, Sialkot, Pakistan) and 27-G long needles (Carpule, Heraeus Kulzer Gmbh, Hanau, Germany). After aspiration at the target area, the solution was injected at a rate of 1 mL/min. After 15 min, the subjects were questioned about the presence of lip numbness and the teeth were re-examined with similar cold pulp sensitivity test and electric pulp test to confirm pulpal anesthesia. In some cases supplementary injections were used, followed by isolation of teeth with a rubber dam and endodontic access cavity preparation. Then the root canal orifices were located. 

All the procedures were performed by one clinician in order to eliminate or minimize interpersonal variability in the treatment procedures. The initial working length was recorded with a #15 K-file (Dentsply Maillefer, Ballaigues, Switzerland) and an apex locator (Root ZX apex locator, J. Morita USA, Inc., Irvine, CA, USA) and confirmed by digital radiography. In both groups the working length was 0.5-1 mm shorter than the length determined using the radiographic technique [18]. The patients were randomly assigned to one of the two treatment groups (*n*=39).

In the RaCe group, RaCe instruments (FKG Dentaire, La-Chaux-de Fonds, Switzerland) were used in crown-down technique according to the manufacturer’s instructions; with the following sequence: 40/0.10 and 35/0.08 for the preparation of the coronal third of each root canal followed by 30/0.06 in the middle third, #25/0.04 in the apical third and #30/0.04 up to the working length. The final apical size was achieved with #30/0.04 or #35/0.04 file. All preparation procedure was done using gentle in-and-out motions and the instruments were pulled out when resistance was encountered and the next instrument in the sequences was used. 

**Table 1:** Inclusion and exclusion criteria

**Inclusion Criteria**	**Exclusion Criteria**
20-50 years of age No systemic diseases Asymptomatic tooth Normal periapical view Restorable teeth Periodontal scoring index <2	Age <20 and >50 Systemic diseases Allergy to lidocaine Inability to take ibuprofen Pregnancy or nursing Symptomatic pulpitis Pulp necrosis PDL widening Periapical radiolucency Sinus tract Periapical abscesses Presence of resorption Tooth malposition Fixed partial dentures Not signing consent Use of analgesics in the last 12 h

In the other group, ProTaper (Dentsply-Maillefer, Ballaigues, Switzerland) instruments were used in the crown-down technique according to the manufacturer’s instructions, with motions similar to those with RaCe instruments. The instrumentation sequence was as follows: SX (auxiliary shaping file, tip size 17) was used to shape the coronal portion of the root canal, followed by S1 (tip size 20) in the coronal third and S2 (tip size 19) in the middle third; in addition, both instruments were used to progressively enlarge the apical third. Finally, the finishing files (F1, 20/0.07 and F2, 25/0.08) were used to finish the apical third. The final apical preparation diameter was matched to K-file #30 or #35, depending on the initial apical size and the canal curvature. 

Concomitant with the use of rotary files for cleaning and shaping of the root canals, gel-form 17% EDTA (Ariadent, Tehran, Iran) was used as a lubricant. During all the preparation procedures with both rotary systems, the root canals were irrigated with 30 mL of normal saline using a syringe connected to a 25-guage needle after each file. The needle was inserted into each root canal as far as possible, without binding. Finally the pulp chamber and the root canals were irrigated with 5 mL of 2.5% sodium hypochlorite solution. After the final rinse with normal saline solution, the root canals were dried with paper points and the standard ISO-sized matching master cones were fitted and checked with radiography. Then the root canals were obturated with gutta-percha (Meta Biomed, Cheongju, Korea) and AH-26 sealer (Dentsply, Tulsa Dental, Tulsa, OK, USA), using the lateral compaction technique. A temporary filling material (Zoliran; Golchai, Tehran, Iran) was placed and the occlusion was checked. Then the patient was referred to the restorative department for the final restoration.

**Figure 1:**
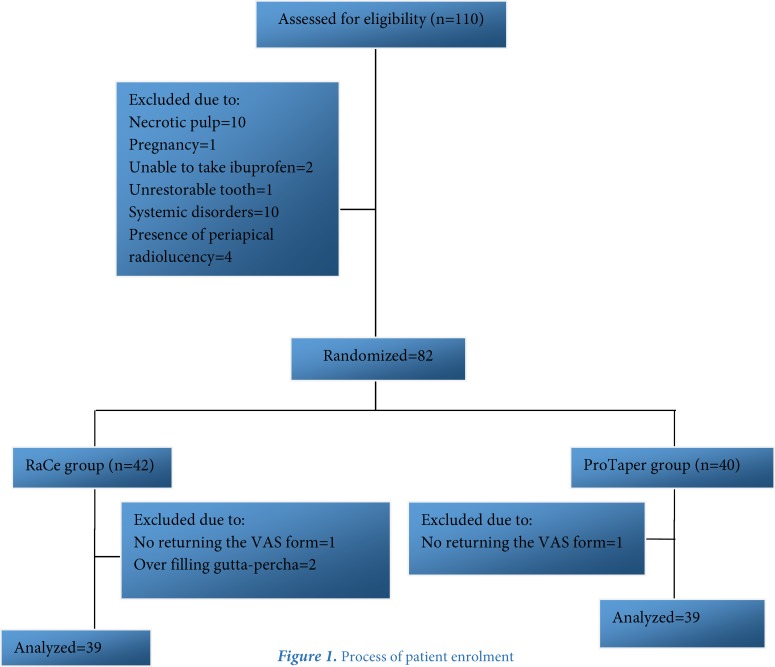
Process of patient enrolment

**Figure 2:**
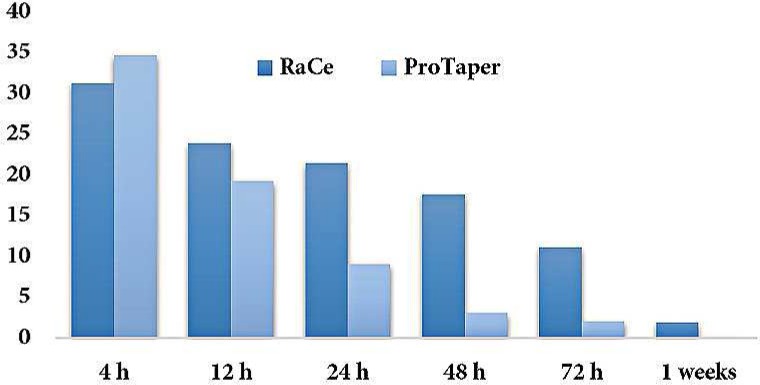
Mean pain severity at 4-, 12-, 24-, 48- and 72-hour and 1-week postoperative intervals in both groups

The patients were calibrated and asked to mark their pain on a visual analogue scale (VAS) at 4-, 12-, 24-, 48- and 72-h and 1-week postoperative intervals. Although no systemic medications were prescribed, the patients were instructed to take mild analgesics (400-mg ibuprofen or Gelofen; Jabberebne Hayyan, Tehran, Iran) in case of pain perception and record it. Since ibuprofen has dose-dependent activity and its analgesic effect completely disappears after 8 h [19], these patients were evaluated at 24,- 48- and 72-h and 1-week intervals similar to other patients in the study. Patients taking more than two tablets of ibuprofen during the first 24 h and those taking any dose of the medicine after 24 h were excluded from the study. 

The VAS was divided into the following 6 categories during data analysis: 0 mm; no pain, 0-20 mm; mild pain, 20-40 mm; moderate pain, 40-60 mm; severe pain, 60-80 mm; very severe pain and 80-100 mm; the worst pain conceivable [20].

The SPSS statistical package (Statistical Package for Social Science, SPSS version 18.0, SPSS, Chicago, IL, USA) was used for statistical analysis. The data were then analyzed with repeated-measures ANOVA; the Mann-Whitney U test was used for two-by-two comparisons. Statistical significance was set at 0.05.

## Results

A total of 82 subjects contributed to this study. Four patients were excluded because of not filling out the VAS forms and over-extrusion of gutta-percha root filling during treatment (Figure 1). In both groups, the pain severity at 4-, 12-, 24-, 48- and 72-h and 1-week postoperative intervals exhibited a significant decrease from the beginning to the end at all intervals (*P*<0.001) (Figure 2) (Table 2).

Comparison of mean pain severity between the two groups at various postoperative intervals did not reveal any significant differences between the two groups (*P*=0.10). 

In this context, the pain severity at 4-h interval in the RaCe group was less than that of the ProTaper group, but the difference was not statistically significant (*P*>0.05). At 12-, 24-, 48- and 72-h and 1-week postoperative intervals, the pain severity in the ProTaper group was less than that of the RaCe group; after 48- and 72-h the difference was significant (*P*<0.001), however, after 12-, 24-h and 1-week intervals, the differences in pain severity between the two groups were not significant (*P*>0.05) (Figure 2) (Table 2).

During the first 24 h, 17% of patients from both groups had no pain and 5% in RaCe group and 2% in ProTaper group had the worst pain conceivable.

The number of patients taking analgesics during the first 24-h postoperative period was 20 (51.3%) and 15 (38.5%) in RaCe and ProTaper groups, respectively, with no significant differences between the two groups (*P*=0.25).

## Discussion

The aim of the present study was to compare the effect of root canal treatment with RaCe and ProTaper rotary files on the intensity of postoperative pain subsequent to endodontic treatment. Based on the results of the present study, comparison of mean pain severity between the two groups at various postoperative intervals did not reveal any significant differences.

The incidence of postoperative pain ranges from 1.4% to 16% [1], with the extrusion of debris into periradicular tissues being reported as the main etiologic factor for periapical inflammation and postoperative pain, which is referred to as a flare-up [21]. The etiologies of flare-up are not always clear; however, fluctuations in periapical tissue pressure, the number or virulence of endodontic microorganisms, or the environmental conditions have been reported as possible reasons [22].

Irritation of periapical tissues results in inflammation and release of many chemical substances which initiate inflammatory responses [23]. The mediators released include neuropeptides, arachidonic acid metabolites, cytokines, lysosomal enzymes, platelet-activating factor, fibrinolytic peptides, vasoactive amines, anaphylatoxins and kinins [23]. Therefore, the amount of debris extruded through the apical foramen into the periapical tissues should be kept to a minimum amount during root canal instrumentation. The amount of extruded debris [24] and neuropeptides released from C-fibers found in the periodontal ligament [25], differ with the use of different instrumentation techniques, which explains the differences in the severity of postoperative pain experienced by patients.

**Table 2:** Mean pain severity at 4-, 12-, 24-, 48- and 72-hour and 1-week postoperative intervals in both groups

**Group** **/Pain severity**	**4 h**	**12 h **	**24 h**	**48 h**	**72 h**	**1 week**
**RaCe**	31.30±4.78	23.89±4.	21.48±4.31	17.61±4.34	11.12±3.05	1.89±1.06
**ProTaper**	34.71±5.11	19.25±3.75	9.07±2.48	3.05±1.92	2.07±1.48	0.12±.012

In this context, techniques employing rotational movements result in the extrusion of less debris compared to techniques employing pull-push movements [26]. NiTi rotary instruments differ in their design, cross-sections and application methods [27], resulting in varying amounts of debris extruded into the periapical tissues [6]. Azar *et al.* [28] compared the quantity of debris and the irrigants extruded apically with the use of ProTaper, ProFile and K-Flexofile instruments and reported that all these systems resulted in the extrusion of debris and irrigants. 

The majority of NiTi rotary instruments are applied in a crown-down technique, in which they first enlarge the coronal third of the root canal to provide a passageway for debris to escape from the root canal (due to Archimedes’ screw effect) which reduces the apical extrusion of debris [9], and this is consistent with the present study.

Investigators suggested that presence of preoperative pain and presence and size of periapical radiolucency are related [2]; in this context, preoperative pain has been used as a good predictor for postoperative pain [2]. Furthermore, some recent studies have reported that some anaerobic bacteria in necrotic pulps are associated with more clinical symptoms [29], leading to the selection of asymptomatic irreversible pulpitis in the present study. In addition, the type of tooth, pulp and periapical status, and the type and the volume of the irrigants used were matched between the two groups in the present study to reduce the confounding variables during the preparation steps, except for the instrument design. 

Based on a study by Seltzer [30], there is a relationship between root canal obturation level and the incidence of postoperative pain, reporting the incidence of postoperative pain to be 14%, 53% and 60% in patients with under-filling, flush-filling and over-filling, respectively. Therefore, in the present study, two patients were excluded because of over-extrusion of gutta-percha.

In the present study, all the subjects underwent one-visit root canal treatment because a meta-analysis showed that patients undergoing one-visit root canal therapy exhibited significantly less endodontic postoperative pain compared with those undergoing a two-visit treatment protocol [31]. 

Cold lateral condensation obturation technique was used in the present study because Alonso-Ezpeleta *et al.* [32] reported that this technique resulted in minimum postoperative pain in comparison with the thermal obturation technique.

Medicines from the NSAID family are usually administered as the first analgesic choice subsequent to root canal treatment, among which ibuprofen is the most commonly used medication [33]. It was reported that regular or on-demand use of 400-mg ibuprofen did not significantly relieve pain [33]. In the present study, ibuprofen was prescribed on an on-demand basis after treatment.

Pain is usually manifested a few hours or days after root canal treatment, necessitating unscheduled visits [1]; therefore, we monitored postoperative pain for one week. Pain perception has a subjective nature and is modulated by multiple physical and psychological factors; therefore, pain measurement is inherently difficult [34]. Different scales and methods have been used to assess pain after endodontic therapy [34]. Visual analogue scale (VAS) is a 100-mm horizontal line, with vertical markings at both ends [34], indicating no pain and the most severe pain conceivable at two ends; no numbers are used between the two ends. In order to quantify the line, a mm-graded ruler is used to measure the length of the horizontal line and determine the numbers indicating pain severity [34] .In the current study VAS was considered as a valid and reliable scale for evaluation of pain perception [34].

Postoperative pain steadily decreased over time. After a day, the mean pain severity exhibited a decrease of 40%. Seven days after treatment, the pain severity decreased to less than 10% [1], consistent with the present study.

Tasdemir *et al*. [15] showed that ProTaper and Mtwo Rotary instruments extruded more debris than RaCe and Garlapati *et al.* [16] reported that Mtwo and ProTaper instruments exhibited significantly more apical extrusion of bacteria than RaCe. Furthermore, Tanalp *et al.* [17] showed that ProTaper systems resulted in significantly greater amounts of extruded debris in comparison with other continuous rotary techniques.

One of the reasons for the absence of significant differences in postoperative pain between the two rotary groups in the present study might be the fact that periapical tissues serve as a natural barrier against extrusion of debris, preventing extension of the *in vitro* results to clinical situations. In addition, there were no significant differences between the two groups regarding the use of analgesics during the first 24-h postoperative interval.

Future research is suggested to compare postoperative pain experienced by patients with symptomatic irreversible pulpitis and pulp necrosis after root canal preparation with RaCe and ProTaper rotary instruments.

## Conclusion

This study showed no significant differences in the postoperative pain experienced by patients between the two rotary groups, indicating the clinical acceptability of both endodontic techniques.
